# Multiple angiosarcomas of both breasts: a case report

**DOI:** 10.1186/s40792-023-01782-w

**Published:** 2023-11-28

**Authors:** Ryota Matsuda, Michiyo Saimura, Keisei Anan, Kento Katsuyama, Yuki Tahara, Kanako Kurata, Kohei Horioka, Mitsuhiro Nakamoto, Kenichiro Koga, Shoshu Mitsuyama, Eiichi Sato, Shinichi Wakamatsu, Sadafumi Tamiya

**Affiliations:** 1 Department of Surgery, Kitakyushu Municipal Medical Center, 2-1-1 Bashaku, Kokurakita-Ward, Kitakyushu, Fukuoka 802-0077 Japan; 2 Department of Medical Oncology, Kitakyushu Municipal Medical Center, 2-1-1 Bashaku, Kokurakita-Ward, Kitakyushu, Fukuoka 802-0077 Japan; 3 Wakamatsu Oncology Clinic, 2nd floor, Kokura KMM Building, 2-14-1 Asano, KokuraKita-Ward, Kitakyushu, Fukuoka 802-0001 Japan; 4 Department of Pathology, Kitakyushu Municipal Medical Center, 2-1-1 Bashaku, Kokurakita-Ward, Kitakyushu, Fukuoka 802-0077 Japan

**Keywords:** Angiosarcoma, Bilateral breasts, Radiotherapy, Chemotherapy, Paclitaxel, Metastasis

## Abstract

**Background:**

Primary angiosarcomas of the breast are rare and highly aggressive. We herein report a rare case of multiple angiosarcomas detected concurrently in both breasts.

**Case presentation:**

A 49-year-old woman visited a doctor after noticing a lump in her right breast. At that time, mammography and ultrasonography revealed no abnormal findings in either breast. She was referred to our hospital 5 months later, because screening mammography had revealed a focal asymmetric density in her right breast. Ultrasonography showed ill-defined hyper- and hypo-echoic lesions in both breasts. Magnetic resonance imaging disclosed five heterogeneously enhanced masses (5.8 cm in maximum diameter) in the right breast and six enhanced masses (approximately 1–3 cm in diameter) in the left breast. Histological examination of core needle biopsies revealed proliferation of irregularly shaped vascular channels lined by atypical endothelial cells throughout the adipose tissue and lobules of the breasts, leading to a diagnosis of well-differentiated angiosarcoma. The lesions were assumed to be primary angiosarcomas, because she had neither a history of breast surgery nor of radiation therapy. She underwent bilateral mastectomies and postoperative chest wall irradiation. Computed tomography 11 weeks after the surgery revealed multiple, small, subcutaneous nodules in the chest wall that were suspected of being angiosarcoma metastases. We started chemotherapy (weekly paclitaxel 80 mg/m^2^), which achieved shrinkage of these nodules within 2 months.

**Conclusions:**

Early diagnosis, immediate initiation of local and systemic therapies, and intensive follow-up are important in improving the prognosis of angiosarcomas.

**Supplementary Information:**

The online version contains supplementary material available at 10.1186/s40792-023-01782-w.

## Background

Primary angiosarcomas of the breast are rare and highly aggressive. They comprise 0.04% of all breast tumors and approximately 8% of breast sarcomas [1]. Because of their rarity and lack of characteristic clinical findings, diagnosis of angiosarcomas can be difficult. These tumors have a high propensity for distant metastasis and the overall prognosis is poor, reported 5-year survival rates being approximately 10% [2]. We herein present a case of multiple angiosarcomas in both breasts.

## Case presentation

A 49-year-old woman visited a doctor, because she had noticed a lump in her right breast. There were no abnormal findings in either breast at that time. She was referred to our hospital 5 months later, because screening mammography revealed a focal asymmetric density (FAD) in her right breast. She had neither a history of breast surgery nor of radiation therapy. She had a family history of cancer: her aunt had breast cancer, and her brother renal cancer.

**Fig. 1:**
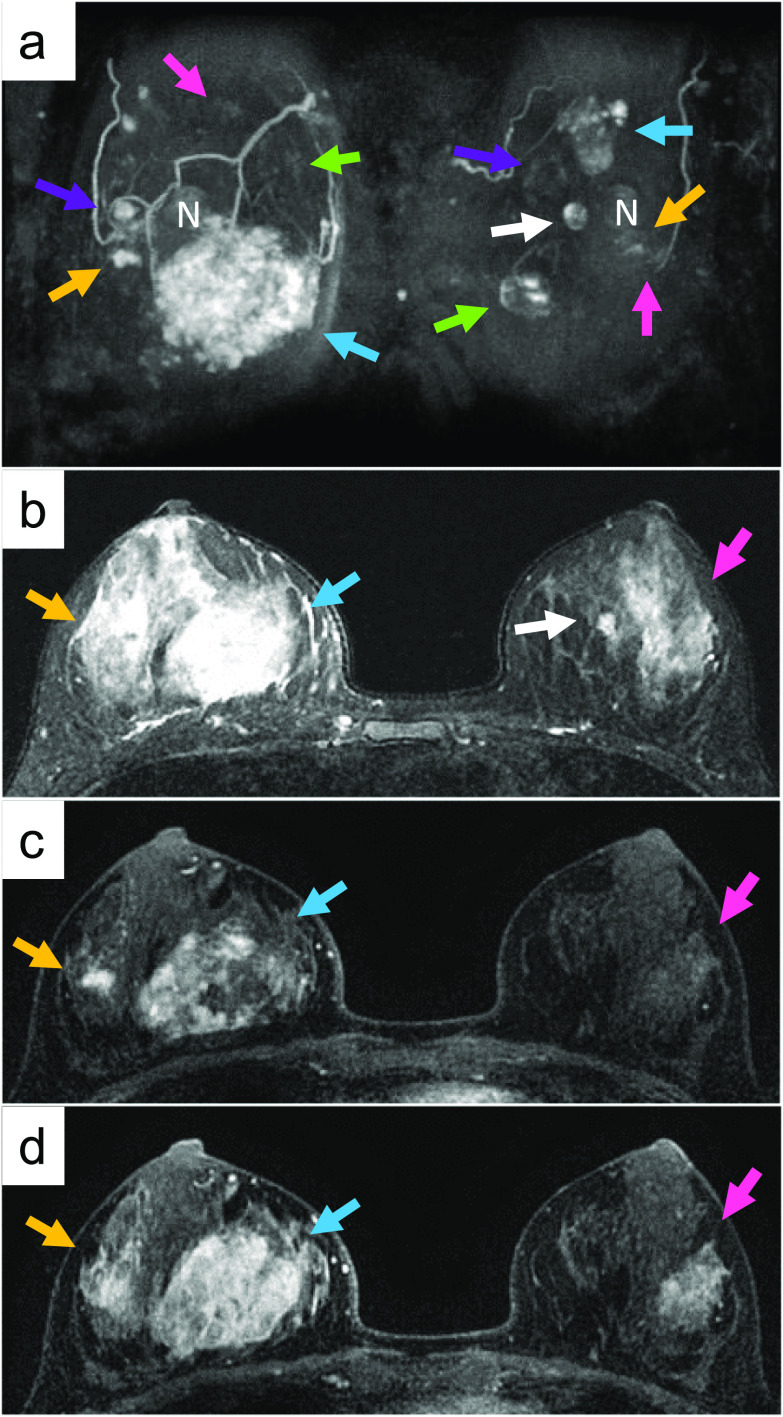
Magnetic resonance imaging showed five heterogeneously enhanced masses, 5.8-cm-diameter at a maximum, in the right breast, and six enhanced masses, approximately 1–3 cm in diameter, in the left breast (arrows in **a**). These masses showed high intensity on fat-suppressed T2-weighted images (**b**). The contrast-enhanced images exhibited heterogeneous enhancement in the early phase (**c**), and progressive and concentric enhancement in the delayed phase (**d**). N: nipple

Physical examination revealed a soft, mobile, 5-cm-diameter mass in her right breast, and a 2.5-cm-diameter mass in her left breast. Mammography showed a FAD in the inferior part of the right breast (Additional file 1: Fig S1). Ultrasonography showed ill-defined hyper- and hypo-echoic lesions in the inner and outer lower quadrants of the right breast and in the inner and outer upper quadrants of the left breast (Additional file 2: Fig S2). Computed tomography (CT) revealed 8- and 3-cm-diameter heterogeneously enhanced masses in the inner and outer lower quadrants of the right breast and several enhanced masses in the left breast (Additional file 3: Fig S3). Magnetic resonance imaging (MRI) showed five heterogeneously enhanced masses (5.8 cm in maximum diameter) in the right breast and six enhanced masses (approximately 1–3 cm in diameter) in the left breast (Fig. 1a). These masses showed high intensity on fat-suppressed T2-weighted images (Fig. 1b). Contrast-enhanced images exhibited heterogeneous enhancement in the early phase (Fig. 1c) and progressive and concentric enhancement in the delayed phase (Fig. 1d). The dynamic enhancement curves showed a persistently enhancing or plateau pattern. Some of the tumors were enhanced in a non-mass-like form and had no well-defined margins. Histological examination of core needle biopsies (CNBs) revealed proliferation of irregularly shaped vascular channels lined by atypical endothelial cells throughout the adipose tissue and lobules of the breasts, leading to a diagnosis of well-differentiated angiosarcoma (Additional file 4: Fig S4). CT scans and bone scintigraphy revealed no metastases. Positron emission tomography–CT showed no 18F-fluorodeoxyglucose uptake in the tumors in either breast, which was considered suggestive of benign mammary lipomas (Additional file 5: Fig S5).

**Fig. 2:**
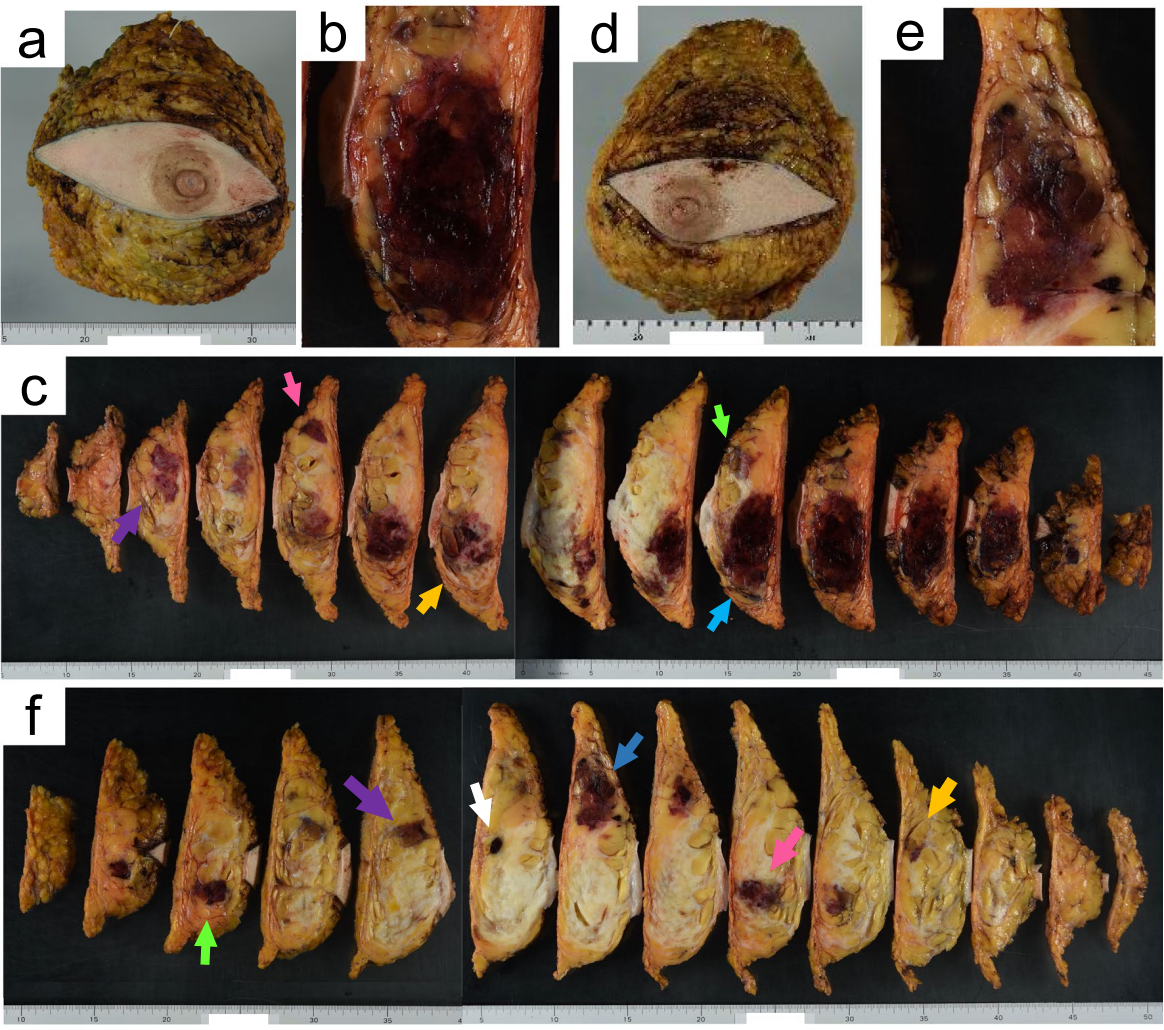
Macroscopically, five masses were observed in the right breast (**a**–**c**, arrows) and six in the left (**d–f**, arrows). The tumors were dark red and hemorrhagic and had diameters of up to 7.5 × 6 cm in the right breast and 5 × 3.5 cm in the left breast

**Fig. 3:**
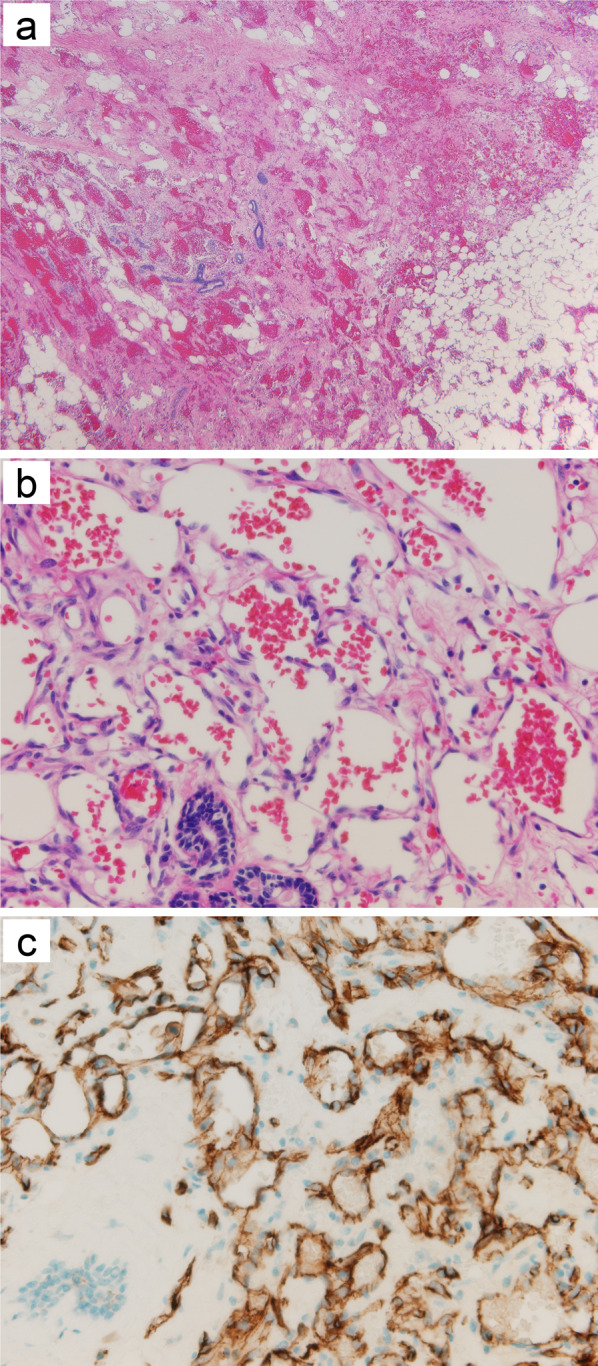
Histological examination of the resected specimens revealed multiple lesions composed of irregularly shaped vascular channels lined by endothelial cells with hyperchromatic nuclei proliferating in the parenchyma and adipose tissue of both breasts (**a**, low power view; **b**, high power view). The tumor cells expressed membranous CD31 immunoreactivity (**c**)

The patient underwent bilateral mastectomy 27 days after her initial visit. We did not perform a sentinel lymph node (LN) biopsy, because there were no clinical findings suggestive of LN metastasis. Macroscopically, five masses were observed in the right breast and six were observed in the left breast (Fig. 2). The tumors were dark red and hemorrhagic and had diameters of up to 7.5 × 6 cm in the right breast and 5 × 3.5 cm in the left. Histological examination revealed multiple lesions composed of irregularly shaped vascular channels lined by endothelial cells with hyperchromatic nuclei proliferating in the parenchyma and adipose tissue of both breasts, (Fig. 3a,b), which expressed membranous CD31 immunoreactivity (Fig. 3c). The tumors were diagnosed as well-differentiated angiosarcomas of both breasts.

**Fig. 4:**
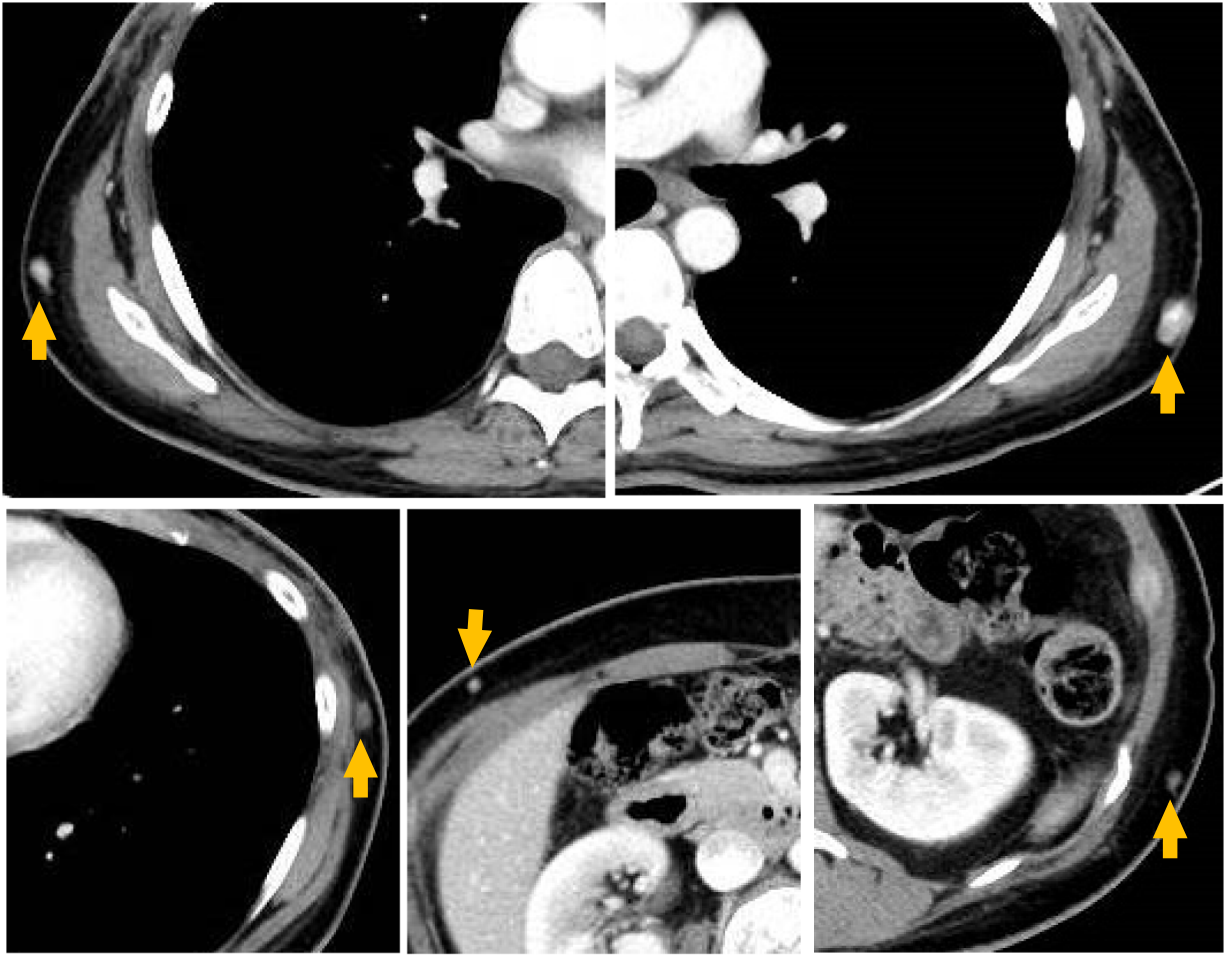
Before adjuvant chemotherapy, 11 weeks after the surgery, computed tomography revealed multiple subcutaneous small nodules on the chest wall outside the irradiation field (arrows). These were suspected of being angiosarcoma metastases

Postoperative irradiation (60 Gy/30 Fr) was administered to the bilateral chest wall. Before adjuvant chemotherapy, 11 weeks after the surgery, CT revealed multiple subcutaneous small nodules on the chest wall outside the irradiation field. These were suspected of being angiosarcoma metastases; however, they were not examined pathologically (Fig. 4). Chemotherapy (weekly paclitaxel 80 mg/m^2^) was started soon thereafter. The nodules shrank within 2 months; further intensive chemotherapy will be administered. Comprehensive genome profiling (FoundationOne®CDx; Foundation Medicine, Cambridge, MA, USA) of the resected specimen detected no reportable alterations with companion diagnostic claims. The microsatellite status was stable, and the tumor mutational burden was 1 mutation/Mb. Other alterations identified were K147E in the *KRAS* gene and V344M-subclonal in the *PIK3CA* gene. These findings yielded no potential targets for targeted therapy.

## Discussion

Primary angiosarcomas of the breast account for less than 0.04% of all breast malignancies, their incidence being approximately 0.0005% [3]. Chen et al. reviewed 87 reported cases. Both breasts were involved in 18 of these patients (21%) [2]. We herein report a patient with multiple angiosarcomas in both breasts.

It is difficult to diagnose angiosarcoma of the breast early. They generally present as rapidly growing, ill-defined masses, breast swelling, or asymmetry of the breasts [4]. Two thirds of these neoplasms measure > 5 cm on presentation [5]. Bilateral tumors may be derived from multifocal origins or by metastasis from a contralateral breast. Mammography often shows non-calcified masses or FADs. Ultrasonography reveals either hyperechoic or mixed hyper- and hypo-echoic masses with architectural distortion. Dynamic contrast-enhanced MRI reveals significant heterogeneous enhancement in the early phase and varying degrees of concentric enhancement in the delayed phase [6]. In the present case, the MRI findings were helpful to distinguish angiosarcomas from typical breast cancers. Well-differentiated angiosarcoma is rich in capillary networks, and the lumens are complete. The contrast agent takes a longer time to pass throughout the tumor and be washed out. Therefore, the dynamic curve exhibits a persistently enhancing or plateau pattern. CNBs are preferred for diagnosing angiosarcomas, because the false negative rate for fine needle aspiration cytology is as high as 40%. It is sometimes challenging for pathologists to diagnose such well-differentiated angiosarcomas, even by CNB, and close collaboration between clinicians and pathologists may help to make an accurate diagnosis. In the current case, the pathologist was well-informed of the patient’s clinical findings, including the MRI results indicative of malignancy, and conclusively diagnosed the bilateral tumors in the CNB specimens as well-differentiated angiosarcomas. That was more than 5 months after the patient first reported detecting a lump in her right breast.

Total mastectomy, with or without radiation therapy, has been the mainstay of treatment. However, locoregional recurrences are reported in half of all patients [7]. There have been no randomized clinical trials assessing the outcomes of breast-conserving surgery versus mastectomy, or determining the optimal margin width after resection [8]. Adjuvant radiation therapy significantly improved recurrence-free survival in a meta-analysis including 380 patients with primary angiosarcoma and 595 with secondary angiosarcoma [9]. In the current case, we performed bilateral total mastectomy, because the tumors were widespread throughout both breasts. Postoperative irradiation was administered to the bilateral chest wall soon after surgery to prevent local recurrences, because most local recurrences occur early after surgery [7]. In addition, McKay et al. reported that low-dose radiotherapy resulted in a rapid and complete response within the treated areas in a patient with recurrent angiosarcoma [10]. These findings suggest that radiation should be more widely considered for the treatment of angiosarcoma. Axillary LN metastases are uncommon at the time of primary therapy; therefore, routine axillary dissection is not indicated [7]. Herb et al. reported that 5% of patients with non-metastatic angiosarcoma of the breast had involvement of regional LNs, and their median overall survival was significantly shorter than that of LN-negative patients (15 months vs. 77 months, log-rank *p* < 0.001) [11]. In the current case, we performed only total mastectomy without axillary clearance, because there were no clinical findings of LN metastasis. No regional LN recurrences were detected 1 year after surgery. A prospective study to determine the indications for sentinel LN biopsy is warranted.

Adjuvant or neoadjuvant chemotherapy may be offered to patients with angiosarcoma of the breast. However, their effectiveness remains controversial and no chemotherapeutic regimens have yet been clearly established. Constantinidou et al. reported the use of a variety of chemotherapy regimens in adjuvant/neoadjuvant settings. The most commonly used regimens in European Organization for Research and Treatment of Cancer sarcoma centers are paclitaxel (27.9%/35.6%, respectively) and gemcitabine and docetaxel-containing regimens (25.6%/11.9%, respectively) [12]. We administered paclitaxel to the present patient, because we suspected that the multiple, small, subcutaneous nodules detected by CT in her chest wall were angiosarcoma metastases. Patients with metastatic angiosarcoma of the breast have had good responses to paclitaxel in a number of studies [7, 13]. According to the National Comprehensive Cancer Network guidelines, there is level 2A evidence that paclitaxel and anthracycline- or gemcitabine-based regimens are the most effective regimens against angiosarcoma [14]. Sher et al. [7] reported an overall response rate of 48% for cytotoxic chemotherapy for metastatic angiosarcoma. Our patient’s small metastases shrank after 2 months of paclitaxel treatment. This relatively high response rate may be partly attributable to the finding that a high proportion of cells in small, fast-growing tumors are killed by doses of drugs that have very little effect on larger, slower-growing masses of otherwise identical cancer cells [15].

Tumor dissemination occurs early via the bloodstream. The most common sites of metastases are bone, lung, liver, contralateral breast, skin, brain, and ovary [2]. Several molecular pathways are reportedly involved in development of angiosarcomas. Loss of function of p53 and overexpression of mouse double minute 2 and vascular endothelial growth factor (VEGF) are alterations in molecular pathways that have been found to be involved in the development of angiosarcomas [16]. The PIK3CA/AKT/mTOR pathway is either directly or indirectly involved in the development of breast angiosarcomas [17]. It has been postulated that restoration of wild-type p53 expression may be an effective therapeutic strategy. PI3K inhibitor and mTOR inhibitor are possible targets for angiosarcoma therapy. A small subset of these tumors shows mutations in *PLCG1* and *KDR*, which are involved in the VEGFR2 signaling pathway [18]. Inhibition of the VEGF pathway is a potentially effective approach to treating angiosarcomas. Comprehensive genome profiling of our patient’s tumor cells did not yield any leads to targeted therapy.

## Conclusions

We reported a case of multiple angiosarcomas in both breasts. It is imperative to clarify the clinical and histological features of breast angiosarcomas to diagnose and treat affected patients promptly and effectively. Considering the disease’s aggressiveness, immediate pathological examination is recommended if there are findings characteristic of angiosarcoma. It is also important to detect recurrence early to improve the prognosis. Further novel strategies for treatment are needed to prolong survival and maintain quality of life.

### Supplementary Information

**Additional file 1. Fig. S1:** Mammography showed a focal asymmetric density in the inferior part of the right breast.

**Additional file 2. Fig. S2:** Ultrasonography showed ill-defined hyper- and hypo-echoic lesions in the inner and outer lower quadrants of the right breast and in the inner and outer upper quadrants of the left breast.

**Additional file 3. Fig. S3:** Computed tomography revealed 8- and 3-cm-diameter heterogeneously enhanced masses in the inner and outer lower quadrants of the right breast and several enhanced masses in the left breast.

**Additional file 4. Fig. S4:** Histological examination of core needle biopsies revealed proliferation of irregularly shaped vascular channels lined by atypical endothelial cells throughout the adipose tissue and lobules of the breasts.

**Additional file 5. Fig. S5:** Positron emission tomography-CT showed no 18F-fluorodeoxyglucose uptake in the tumors in either breast.

## Data Availability

Not applicable.

## Abbreviations

FAD: Focal asymmetric density
CT: Computed tomography
CNB: Core needle biopsy
MRI: Magnetic resonance imaging
LN: Lymph node
VEGF: Vascular endothelial growth factor

## References

[1] Donnell RM, Rosen PP, Lieberman PH, Kaufman RJ, Kay S, Braun DW (1981). Angiosarcoma and other vascular tumors of the breast. Am J Surg Pathol.

[2] Chen KTK, Kirkegaard DD, Bocian JJ (1980). Angiosarcoma of the breast. Cancer.

[3] Strobbe LJA, Peterse HL, Van Tinteren H, Wijnmaalen A, Rutgers EJT (1998). Angiosarcoma of the breast after conservation therapy for invasive cancer, the incidence and outcome. An unforeseen sequela. Breast Cancer Res Treat.

[4] Yang WT, Hennessy BTJ, Dryden MJ, Valero V, Hunt KK, Krishnamurthy S (2007). Mammary angiosarcomas: imaging findings in 24 patients. Radiology.

[5] Gervais M-K, Burtenshaw SM, Maxwell J, Dickson BC, Catton CN, Blackstein M (2017). Clinical outcomes in breast angiosarcoma patients: a rare tumor with unique challenges. J Surg Oncol.

[6] Wu W, Ji Q, Li Z, Wang Q, Liu S, Yu J (2019). Mammography and MRI manifestations of breast angiosarcoma. BMC Womens Health.

[7] Sher T, Hennessy BT, Valero V, Broglio K, Woodward WA, Trent J (2007). Primary angiosarcomas of the breast. Cancer.

[8] Antonescu CR, Hunt KK, Rowe JJ TK. Breast Tumours. In: WHO Classification of Tumours Editorial Board, editor. WHO Classif Tumours. 5th ed. IARC Press; 2019. p. 200–1.

[9] Abdou Y, Elkhanany A, Attwood K, Ji W, Takabe K, Opyrchal M (2019). Primary and secondary breast angiosarcoma: single center report and a meta-analysis. Breast Cancer Res Treat.

[10] McKay MJ, Rady K, McKay TM, McKay JN (2017). A radiation-induced and radiation-sensitive, delayed onset angiosarcoma arising in a precursor lymphangioendothelioma. Ann Transl Med.

[11] Herb J, Maduekwe UN, Goel N, Rosenberger LH, Spanheimer PM (2022). Does angiosarcoma of the breast need nodal staging?. J Am Coll Surg.

[12] Constantinidou A, Sauve N, Stacchiotti S, Blay J-Y, Vincenzi B, Grignani G (2020). Evaluation of the use and efficacy of (neo)adjuvant chemotherapy in angiosarcoma: a multicentre study. ESMO Open..

[13] Nascimento AF, Raut CP, Fletcher CDM (2008). Primary angiosarcoma of the breast: clinicopathologic analysis of 49 cases, suggesting that grade is not prognostic. Am J Surg Pathol.

[14] NCCN Clinical Practice Guidelines in Oncology (NCCN Guidelines®). NCCN Guidelines Version 2.2022 Soft Tissue Sarcoma. 2022. https://www.nccn.org/professionals/physician_gls/pdf/sarcoma.pdf

[15] Norton L (2014). Cancer log-kill revisited. Am Soc Clin Oncol Educ B.

[16] Hirata A, Tsukamoto T, Yamamoto M, Sakai H, Yanai T, Masegi T (2006). Organ-specific susceptibility of p53 knockout mice to N-bis(2-hydroxypropyl)nitrosamine carcinogenesis. Cancer Lett.

[17] Lahat G, Dhuka AR, Hallevi H, Xiao L, Zou C, Smith KD (2010). Angiosarcoma: clinical and molecular insights. Ann Surg.

[18] Huang S, Zhang L, Sung Y, Chen C (2016). Recurrent CIC gene abnormalities in angiosarcomas: a molecular study of 120 cases with concurrent investigation of PLCG1, KDR, MYC, and FLT4 gene alterations. Am J Surg Pathol.

