# Supplementary figures and images for: Multiple angiosarcomas of both breasts: a case report
Source: Surg Case Rep. 2023 Nov 28;9:205. doi: 10.1186/s40792-023-01782-w (PMC10684844; doi:10.1186/s40792-023-01782-w)

## Slide 1
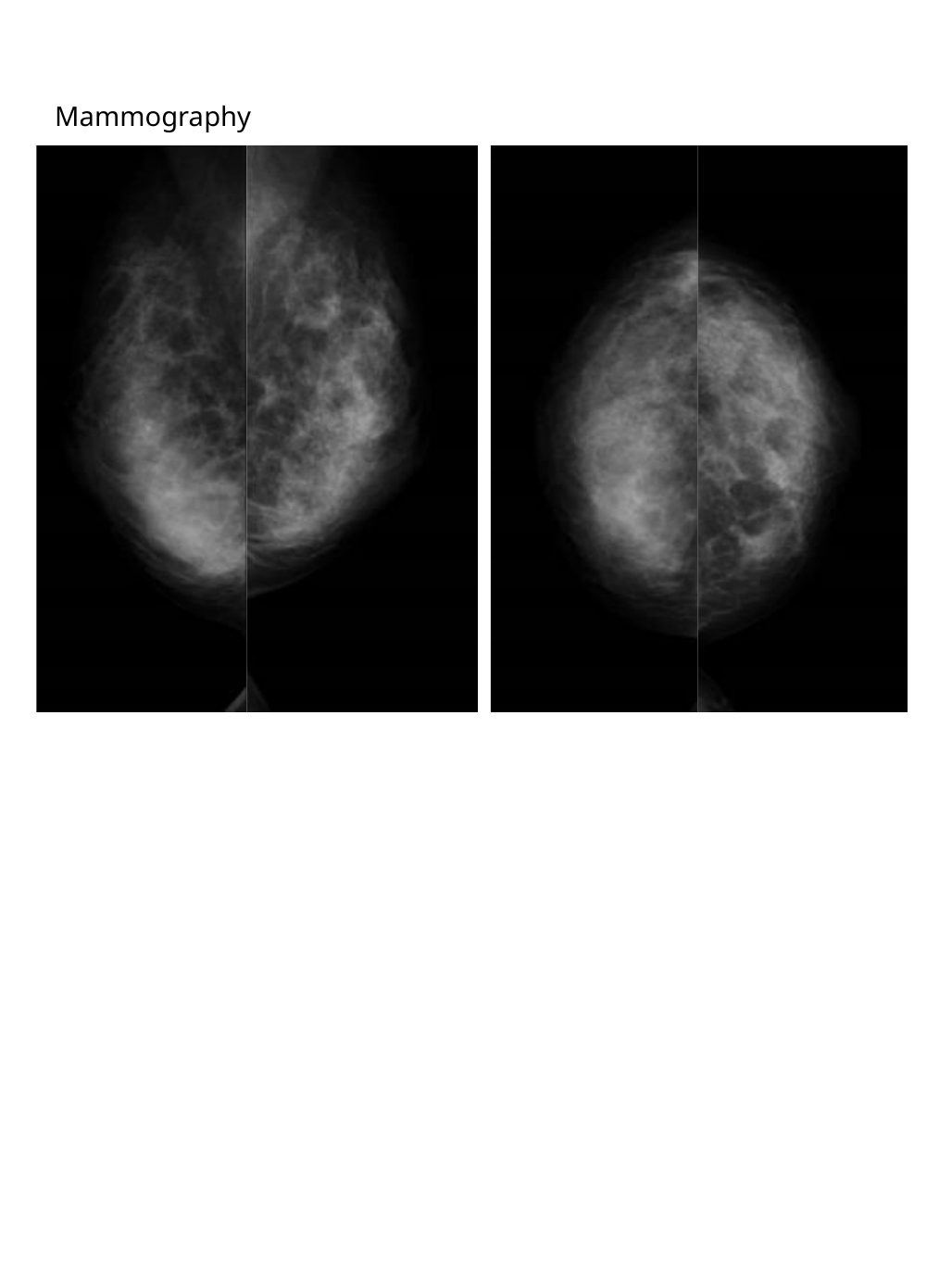

Mammography

Supplement: Supplementary file 1 — Additional file 1. Fig. S1: Mammography showed a focal asymmetric density in the inferior part of the right breast. [file 40792_2023_1782_MOESM1_ESM.pptx]

## Slide 1
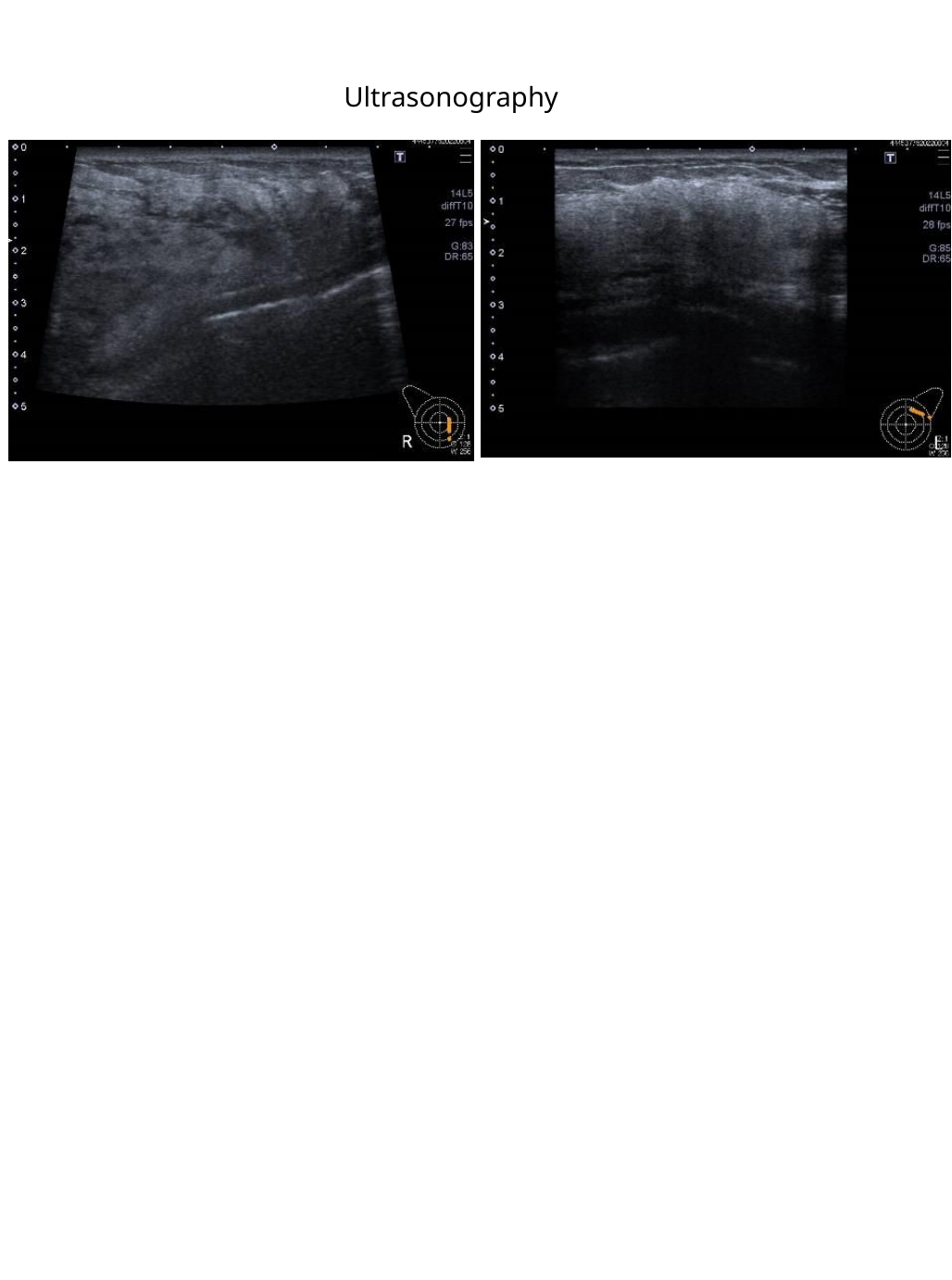

Ultrasonography

Supplement: Supplementary file 2 — Additional file 2. Fig. S2: Ultrasonography showed ill-defined hyper- and hypo-echoic lesions in the inner and outer lower quadrants of the right breast and in the inner and outer upper quadrants of the left breast. [file 40792_2023_1782_MOESM2_ESM.pptx]

## Slide 1
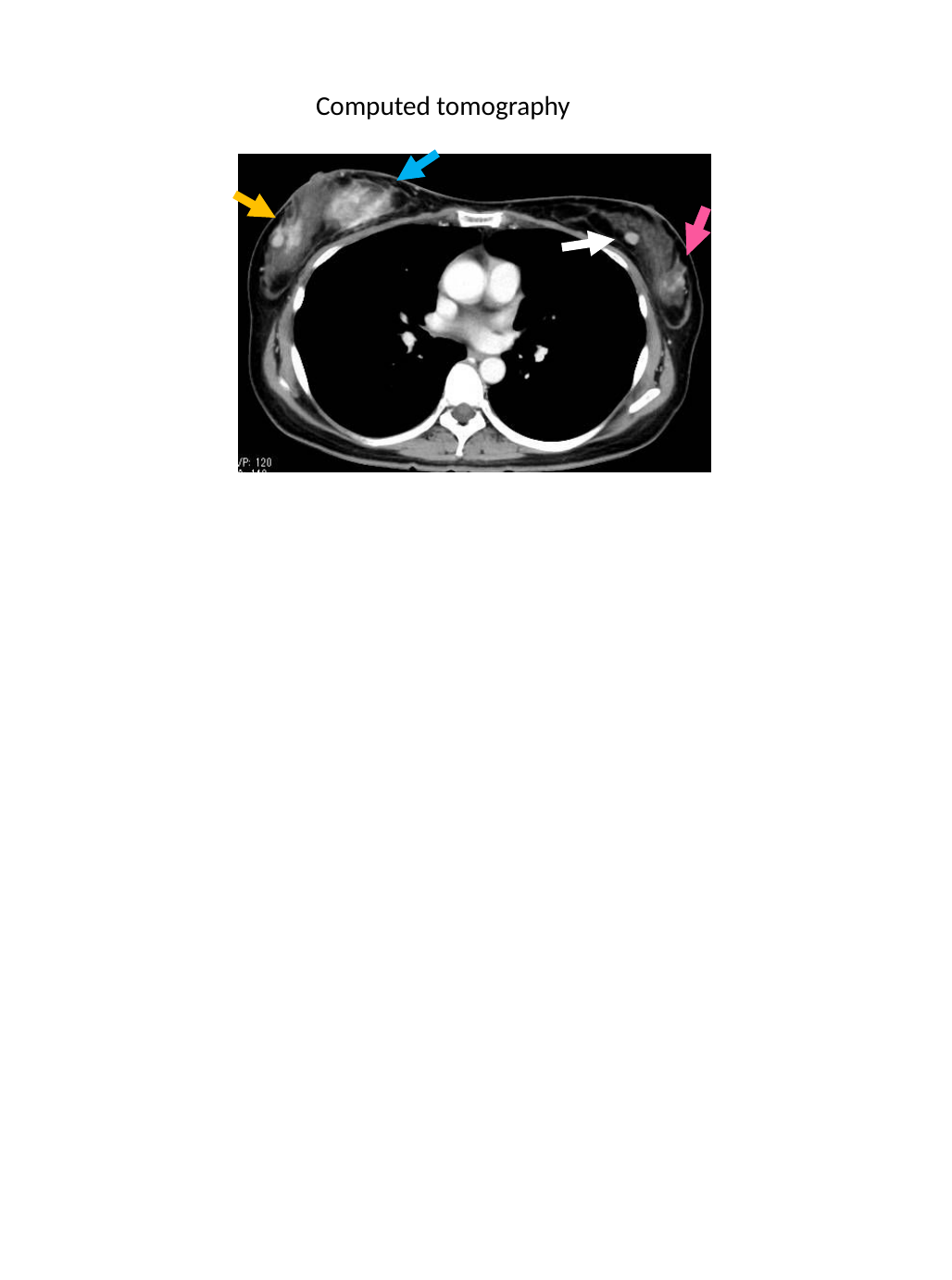

Computed tomography

Supplement: Supplementary file 3 — Additional file 3. Fig. S3: Computed tomography revealed 8- and 3-cm-diameter heterogeneously enhanced masses in the inner and outer lower quadrants of the right breast and several enhanced masses in the left breast. [file 40792_2023_1782_MOESM3_ESM.pptx]

## Slide 1
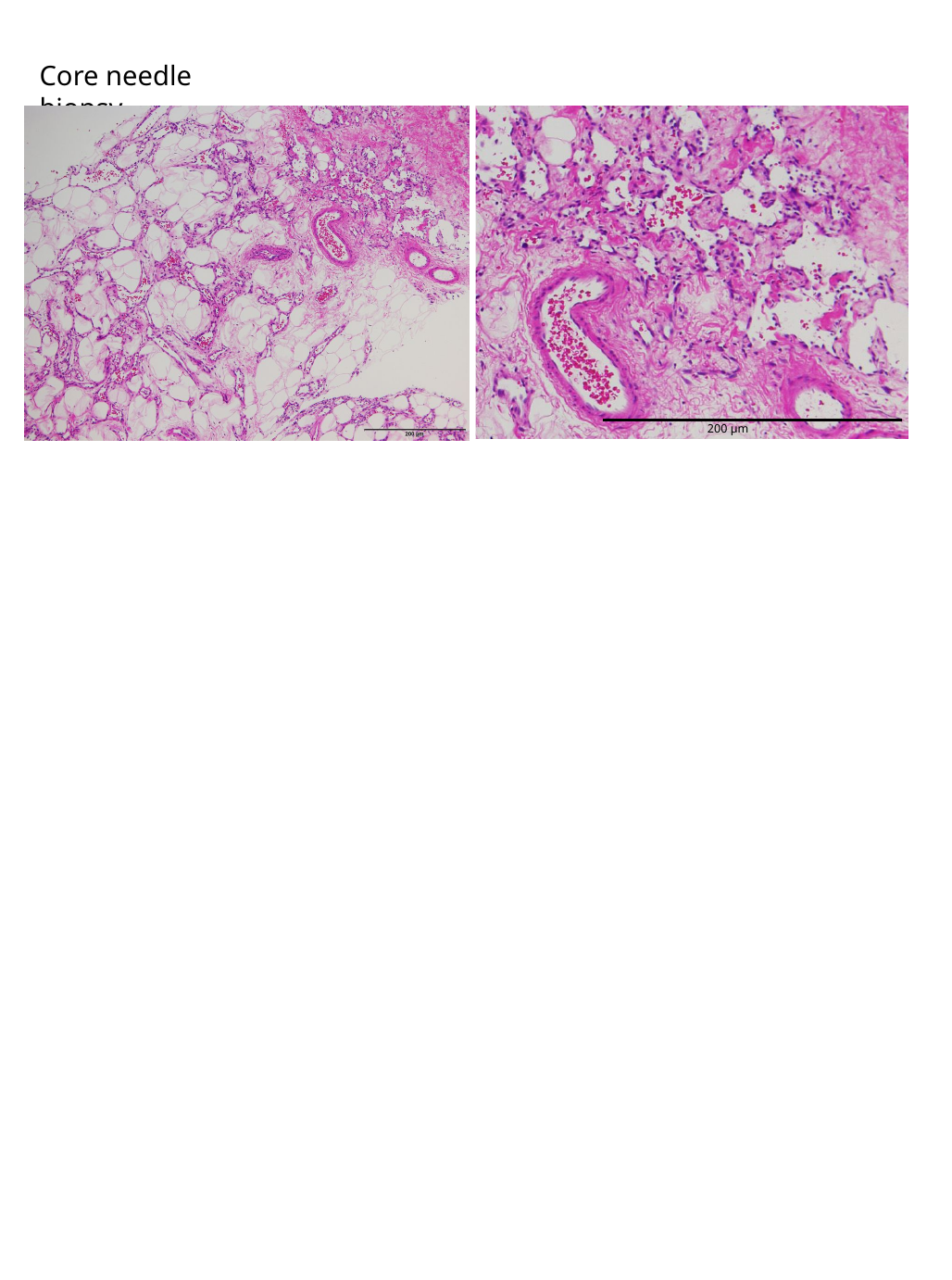

Core needle biopsy
200 μm

Supplement: Supplementary file 4 — Additional file 4. Fig. S4: Histological examination of core needle biopsies revealed proliferation of irregularly shaped vascular channels lined by atypical endothelial cells throughout the adipose tissue and lobules of the breasts. [file 40792_2023_1782_MOESM4_ESM.pptx]

## Slide 1
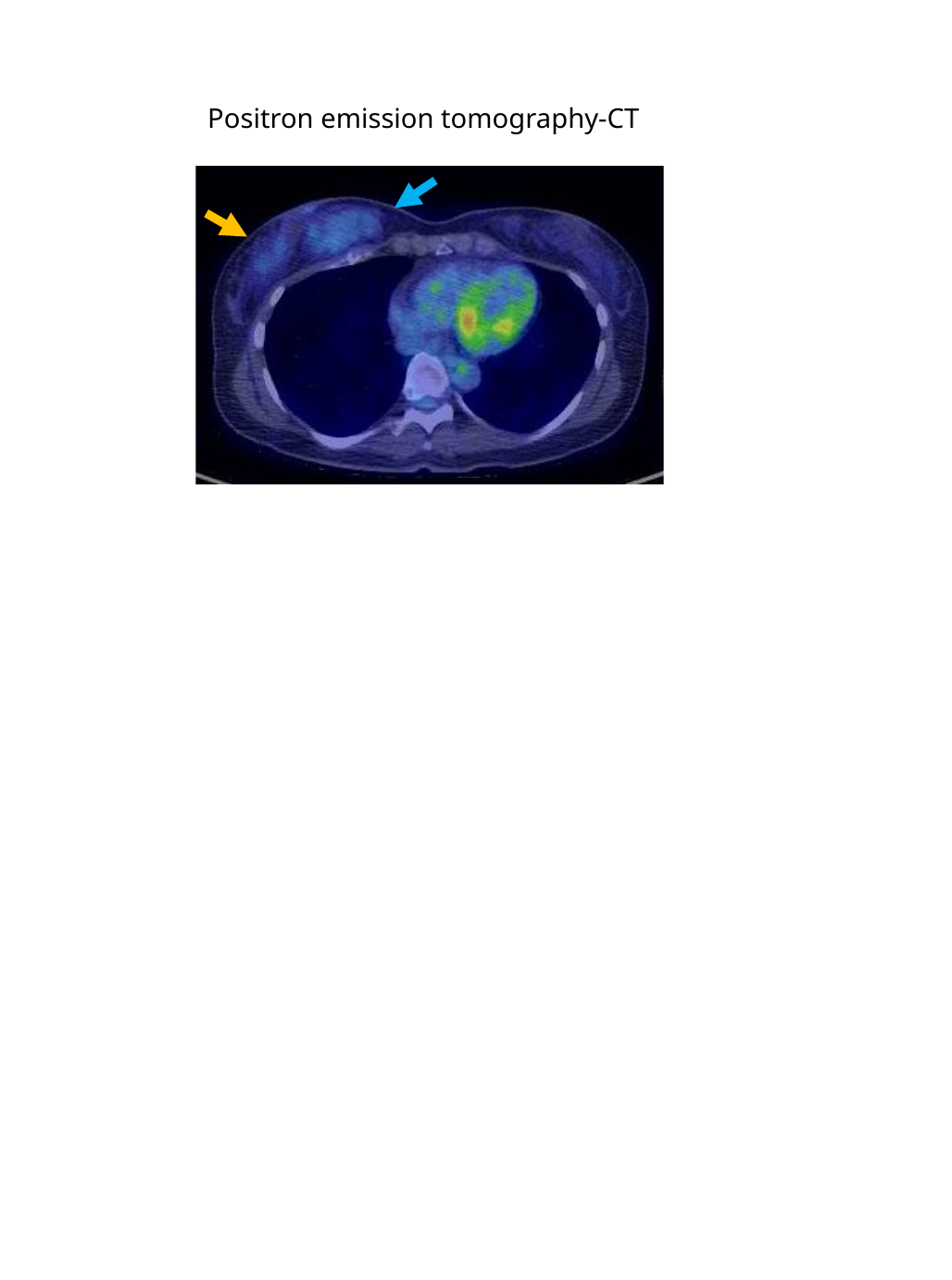

Positron emission tomography-CT

Supplement: Supplementary file 5 — Additional file 5. Fig. S5: Positron emission tomography-CT showed no 18F-fluorodeoxyglucose uptake in the tumors in either breast. [file 40792_2023_1782_MOESM5_ESM.pptx]
